# Structure, substrate recognition and reactivity of *Leishmania major *mevalonate kinase

**DOI:** 10.1186/1472-6807-7-20

**Published:** 2007-03-30

**Authors:** Tanja Sgraja, Terry K Smith, William N Hunter

**Affiliations:** 1 Division of Biological Chemistry and Molecular Microbiology, School of Life Sciences, University of Dundee, Dundee, DD1 5EH, UK

## Abstract

**Background:**

Isoprenoid precursor synthesis via the mevalonate route in humans and pathogenic trypanosomatids is an important metabolic pathway. There is however, only limited information available on the structure and reactivity of the component enzymes in trypanosomatids. Since isoprenoid biosynthesis is essential for trypanosomatid viability and may provide new targets for therapeutic intervention it is important to characterize the pathway components.

**Results:**

Putative mevalonate kinase encoding genes from *Leishmania major *(*Lm*MK) and *Trypanosoma brucei *(*Tb*MK) have been cloned, over-expressed in and proteins isolated from procyclic-form *T. brucei*. A highly sensitive radioactive assay was developed and shows ATP-dependent phosphorylation of mevalonate. Apo and (*R*)-mevalonate bound crystal structures of *Lm*MK, from a bacterial expression system, have been determined to high resolution providing, for the first time, information concerning binding of mevalonate to an MK. The mevalonate binds in a deep cavity lined by highly conserved residues. His25 is key for binding and for discrimination of (*R*)- over (*S*)-mevalonate, with the main chain amide interacting with the C3 hydroxyl group of (*R*)-mevalonate, and the side chain contributing, together with Val202 and Thr283, to the construction of a hydrophobic binding site for the C3 methyl substituent. The C5 hydroxyl, where phosphorylation occurs, points towards catalytic residues, Lys18 and Asp155. The activity of *Lm*MK was significantly reduced compared to MK from other species and we were unable to obtain ATP-binding data. Comparisons with the rat MK:ATP complex were used to investigate how this substrate might bind. In *Lm*MK, helix α2 and the preceding polypeptide adopt a conformation, not seen in related kinase structures, impeding access to the nucleotide triphosphate binding site suggesting that a conformational rearrangement is required to allow ATP binding.

**Conclusion:**

Our new structural information, consistent with data on homologous enzymes allows a detailed description of how mevalonate is recognized and positioned for catalysis in MK. The mevalonate-binding site is highly conserved yet the ATP-binding site is structurally distinct in *Lm*MK. We are unable to provide a definitive explanation for the low activity of recombinant protein isolated from a bacterial expression system compared to material isolated from procyclic-form *Trypanosoma brucei*.

## Background

The biosynthesis of the isoprenoid precursors isopentenyl pyrophosphate (IPP) and dimethylallyl pyrophosphate (DMAPP) is essential for prokaryotic and eukaryotic organisms. These ubiquitous compounds are utilized in the construction of numerous natural products including dolichols, sterols, terpenes and ubiquinones which contribute to myriad biological functions including hormone-based signaling, electron transport in respiration, apoptosis, meiosis and photosynthesis [1]. Two distinct pathways have evolved to provide a pool of the precursors. In plant chloroplasts, algae, most eubacteria and apicomplexans, IPP and DMAPP are synthesized through the deoxyxylulose phosphate (DOXP) pathway, so named after an intermediate [2-4]. Alternatively, the mevalonate (MVA) pathway supplies these precursors in eukaryotes, archaea, a few eubacteria, in the cytosol of plants and of particular interest to us parasites of the genus *Trypanosoma *and *Leishmania *[4,5].

The MVA pathway starts with condensation of three acetyl-CoA molecules to form 3-hydroxy-3-methylglutaryl-CoA (HMG-CoA), which is reduced to (*R*)-MVA by HMG-CoA reductase. Next, two kinases phosphorylate (*R*)-MVA to (*R*)-MVA 5-diphosphate. This compound is subsequently decarboxylated to IPP and IPP isomerase then produces DMAPP from some of the IPP pool. The two phosphorylation steps and the decarboxylation are carried out in ATP-dependent reactions by the structurally related mevalonate kinase (MK), phosphomevalonate kinase (PMK) and mevalonate 5-diphosphate decarboxylase (MDD) respectively.

We have initiated studies of MVA pathway enzymes in protozoan trypanosomatids, parasites that cause severe diseases of humans and livestock. In Africa, *T. brucei *infection results in sleeping sickness while in South and Central America, *T. cruzi *causes Chagas' disease [6]. Protozoans of the genus *Leishmania*, found throughout tropical and sub-tropical areas, are responsible for cutaneous, mucocutaneous and visceral forms of disease [7]. Visceral leishmaniasis and trypanosomiasis are fatal if untreated and since the availability of effective drugs is limited, there is an urgent need to develop improved therapies. In support of such an effort it is important to achieve a comprehensive understanding of parasite metabolism, and to delineate aspects that are similar to the host from those that, by virtue of significant differences, might provide therapeutic opportunities.

**Figure 1:**
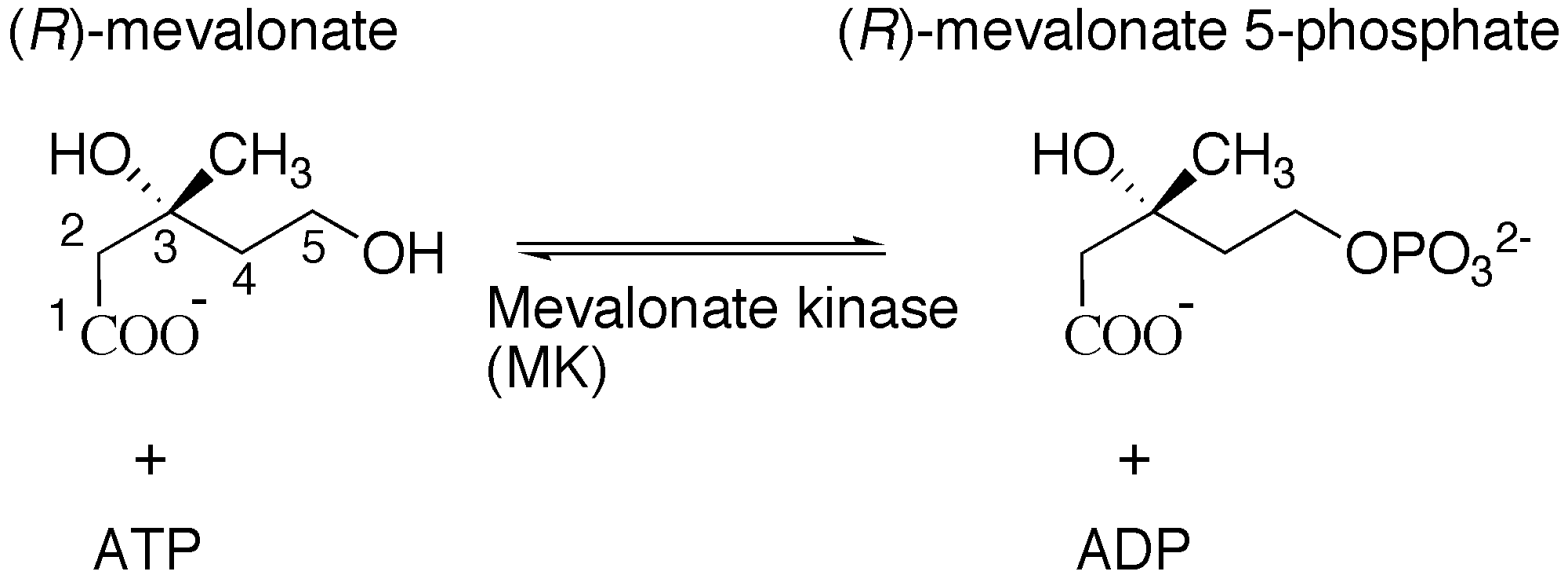
The reaction catalyzed by mevalonate kinase.

We set out to investigate the putative assignment of MK (EC 2.7.1.36) in trypanosomatid parasites. MK catalyzes the fourth step in the MVA pathway, the transfer of the ATP γ-phosphoryl onto (*R*)-MVA to give (*R*)-MVA 5-phosphate (Figure 1) [8-10]. The enzyme is, along with galactokinase (GK), homoserine kinase (HSK), MK and PMK, a founding member of the GHMP kinase superfamily [11]. Family members now also include 4-diphosphocytidyl-2C-methylerythritol (CDPME) kinase, MDD and the archaeal shikimate kinase [12-14]. Structures of MK are known including the apo-structure of *Methanococcus jannaschii *MK (*Mj*MK) and the ATP bound *Rattus norvegicus *MK (*Rn*MK) [8,9].

First it was important to prove that the putative genes do indeed encode a functional MK. Assay of *L. major *MK (*Lm*MK) and *T. brucei *MK (*Tb*MK) derived from procyclic-form *T. brucei *show enzyme activity, and at a greatly enhanced level compared to *Lm*MK derived from a bacterial expression system. High-resolution crystal structures of *Lm*MK are reported and provide, for the first time, insight into the binding of substrate by any MK. There are significant structural differences at the ATP- binding site compared to other GHMP kinases. Consideration of previously published kinetic data on MK, derived from different species, and comparisons allows us to describe generic aspects of MK specificity and mechanism.

## Results and discussion

### Enzyme activity of *Lm*MK and *Tb*MK

The gene assigned as encoding a putative *Lm*MK was cloned into an *Escherichia coli *expression system and yielded 30 mg/L of purified protein. A coupled spectrophotometric assay has been successfully applied to analyze wild type and mutant MK enzymes from different species [10,15-18]. However, no activity was observed for recombinant *Lm*MK using this method although the control experiment with *Mj*MK agreed well with literature values (data not shown). In a further experiment, the fluorescent ATP analogue 2'(3')-O-(2,4,6-trinitrophenyl) adenosine 5'-triphosphate (TNP-ATP) was used to investigate ATP binding following an established protocol [19]. In the presence of *Lm*MK, however, no enhancement in fluorescence could be detected compared to free TNP-ATP indicating that the derivative does not effectively bind the recombinant *Lm*MK (data not shown). This observation is consistent with the conformation of the ATP-binding site observed in the crystal structure and will be discussed later.

We considered the possibility that recombinant *Lm*MK activity might be too low for the spectrophotometric assay and so developed a more sensitive, radioactive assay. Activity of *Lm*MK was observed after incubation of the enzyme with radiolabeled [^3^H] (*R*)-MVA and ATP. After two hours, 22 pmoles of the 83.3 pmoles of [^3^H] (*R*)-MVA present were converted to [^3^H] (*R*)*-MVA *5-phosphate, a turnover rate of 26 % or 1.6 pmol/min/mg.

**Figure 2:**
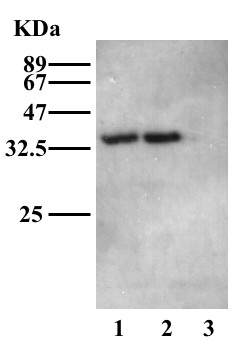
The over-expression of *Tb*MK and *Lm*MK in procyclic-form *T.brucei*. *TbMK *and *LmMK *were cloned into the pLew82 vector and transformed into procyclic *T. brucei *to express ectopic C-terminal HA-tagged proteins under tetracycline control. Mid-log procyclic cells over-expressing *Tb*MK-HA (lane1), *Lm*MK-HA (lane 2) and wild type (lane 3) were washed, lysed and run on an SDS/10 % polyacrylamide gel and transferred to an ECL-Nylon membrane by Western blotting and detected with anti-HA antibodies. The expected molecular weight of both *Tb*MK-HA and *Lm*MK-HA is 36.9 kDa.

Next, *Lm*MK and *Tb*MK were cloned, over expressed in procyclic-form *T. brucei *and immunoprecipitated (Figure 2) prior to undertaking the radioactive assay with each enzyme. A 100-fold lower protein concentration of *Lm*MK and *Tb*MK was assayed, resulting in 2.4 pmoles and 2.7 pmoles of substrate being transformed to product, respectively. This corresponds to turnover rates of 2.9 % (18 pmol/min/mg) and 3.3 % (20 pmol/min/mg), respectively. The radioactive assays prove for the first time, an ATP-dependent MK activity in *Leishmania *and *Trypanosoma *and therefore that the putative genes do indeed encode MK. There is a consistent level of MK activity for the enzymes isolated from procyclic-form *T. brucei *and these enzymes are an order of magnitude more active than the bacterially expressed enzyme. One possible explanation for this difference is that the eukaryotic protein is not optimally folded when produced in *E. coli*. Other possibilities are that a parasite-specific factor might influence MK efficiency, for example, post-translational modification, or some associated parasite protein. Alternatively, that some bacterial specific factor might compromise *Lm*MK activity. However, we have no evidence to support post-translational modification or the presence of additional protein or small molecule species.

### Overall structure

Recombinant *Lm*MK and a selenomethionine (SeMet) derivative produced ordered, isomorphous monoclinic crystals and a single-wavelength anomalous dispersion (SAD) experiment provided initial phase information to solve the structure. The SAD approach was required because molecular replacement calculations using known MK structures failed. Although the non-hydrolysable ATP analogue adenosine 5'-(β,γ-imido) triphosphate (AMPPNP) and (*R/S*)-MVA were included in crystallization solutions there was no indication of ordered ligand binding. A complex was subsequently obtained by soaking crystals in higher concentrations of (*R/S*)-MVA. Here, we describe the high-resolution structures of apo-SeMet *Lm*MK and the binary complex of native *Lm*MK with (*R*)-MVA present in one active site. Crystallographic statistics are presented in Table 1.

There are two molecules in the asymmetric unit (A and B) related by a non-crystallographic (NCS) two-fold axis of symmetry and the surface area between the two is 930 Å^2 ^per molecule, only 6% of the total surface area of the protein. Such a low value is consistent with results from gel filtration and ultracentrifugation experiments that indicate *Lm*MK is a monomer in solution. Molecules A and B overlay with an r.m.s.d. of 1.2 Å for all atoms. Minor differences are observed in the conformation of the three C-terminal residues, several flexible residues at the N-terminal end of α6 and loop regions following β5 and α2. Molecule A of *Lm*MK binds the substrate and overlays on the apo-enzyme molecule A with an r.m.s.d. of 0.7 Å for all atoms. This indicates that there no large-scale conformational changes result from substrate binding and the molecules are so similar that it is only necessary to detail molecule A.

That *Lm*MK presents a monomer in solution makes it different from rat and human MK, which are reported to be dimeric [9,10]. The crystal structure of the rat enzyme has a monomer in the asymmetric unit and a crystallographic two-fold axis of symmetry generates an extended dimer [9]. The alignment of the two molecules in the asymmetric unit of *Lm*MK is similar to that of the rat enzyme in that an extended structure results. However, the orientation of the molecules is very different (not shown).

### The GHMP kinase fold and three conserved motifs

**Figure 3:**
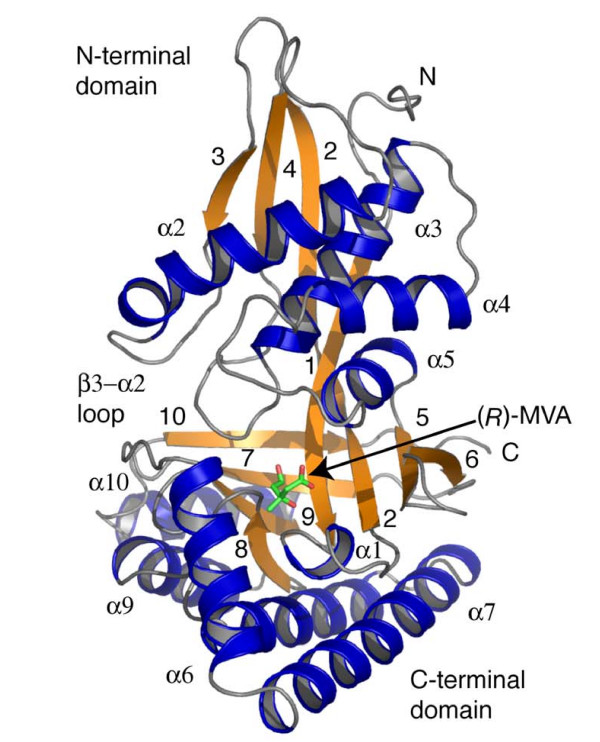
Fold of *Lm*MK. Overall structure of *Lm*MK in complex with (*R*)-MVA. Elements of secondary structure are labeled, helices as αn, and strands numbered, the N and C-termini are marked. The substrate is depicted in a stick representation colored according to atom type (C green, O red).

*Lm*MK exhibits the characteristic GHMP kinase fold consisting of two domains [20]. The fold and the correlation of sequence and secondary structure are presented in Figure 3. The N-terminal domain (residues 1–179) is formed around a six-stranded β-sheet (β1–β6). A small helical segment (α1) is inserted between β1 and β2 and four helices (α2–α5) are positioned on one side of the β-sheet. In the GHMP family this domain is primarily responsible for ATP binding. The C-terminal domain (residues 180–329) contains an anti-parallel four-stranded β-sheet (β7–β10), bordering one end of the N-terminal domain β-sheet, placed on a structure created by five helices (α6-α10).

**Figure 4:**
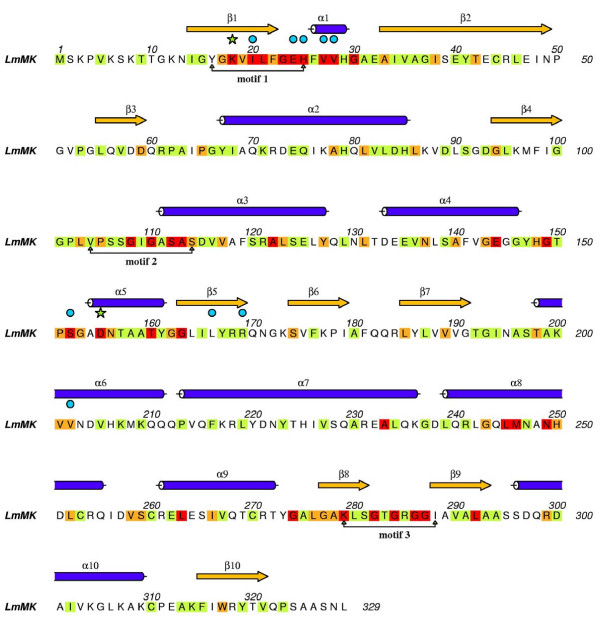
Secondary structure and sequence of *Lm*MK. Invariant or well-conserved residues are boxed in red, orange and lime. Red signifies residues conserved in the three trypanosomatid, *Rn*MK and *Mj*MK sequences, orange residues conserved in any four out of the five sequences, green those conserved in the parasite enzymes. Residues involved in substrate binding are marked with cyan dots and the catalytic aspartate and lysine residues with green stars. The secondary structural elements, as shown in part A, are given above the sequence. Three highly conserved GHMP motifs are marked.

*Lm*MK shares a high sequence identity with *Tb*MK (57 %) and *T. cruzi *MK (*Tc*MK; 60 %, Figure 4) and the structure of *Lm*MK is taken to be representative of trypanosomatid MK. The identity with homologues from archaea and mammals falls below 30 % (data not shown) and the human and rat enzymes are increased in length by about 65 residues. The program DALI [21] identified *Mj*MK (Z-Score: 32.1)^# ^and *Rn*MK (Z-Score: 28.4) as most similar structures to *Lm*MK. The Cα atoms of these structures overlay on *Lm*MK with an r.m.s.d. of 1.7 and 2.1 Å, respectively (not shown).

**Figure 5:**
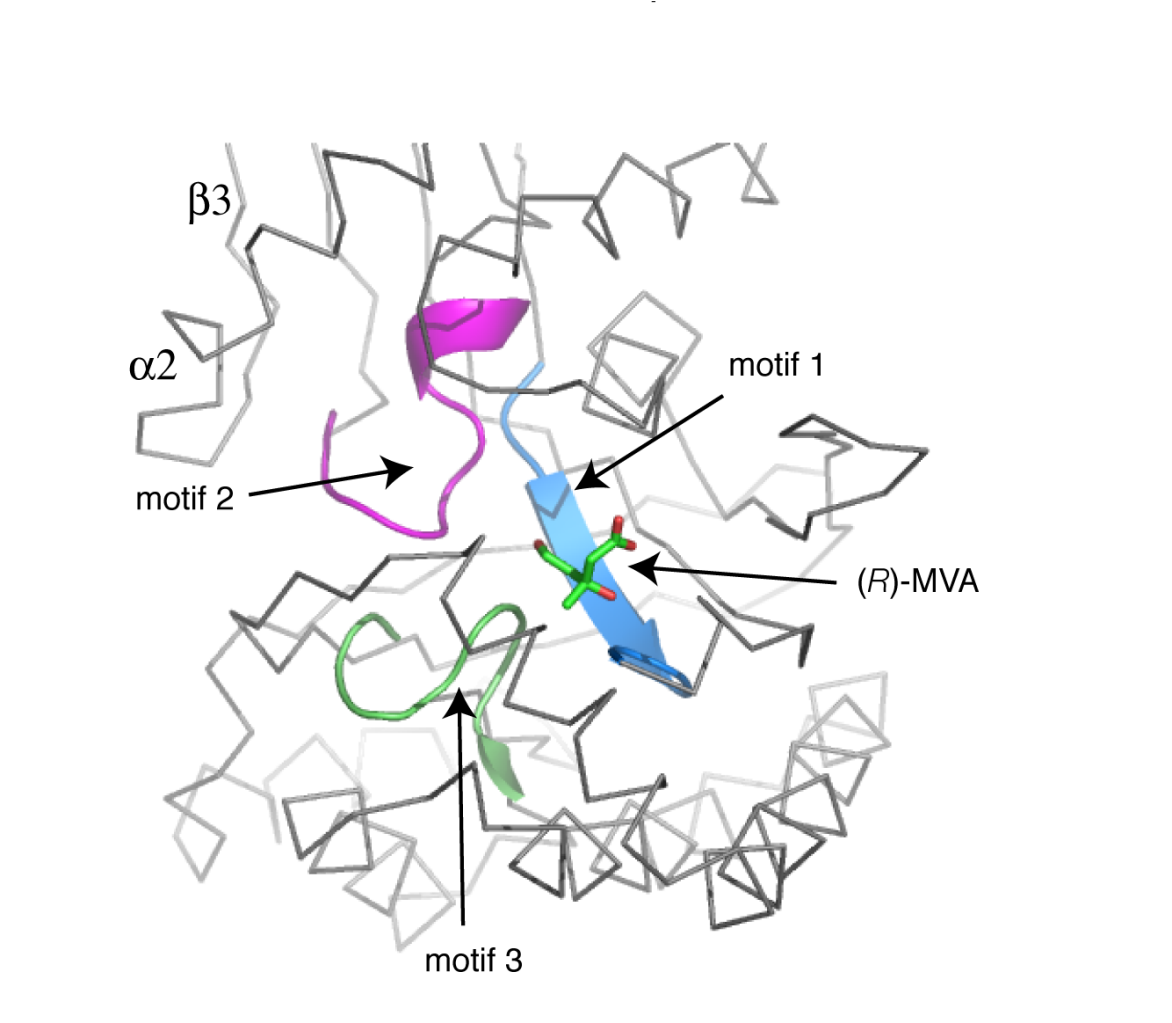
The position of the three conserved GHMP motifs in *Lm*MK. A Cα trace (grey) around the active site is shown and substrate included (shown in stick-mode with C green, O red). Motifs 1, 2 and 3 are shown as ribbons colored cyan, purple and green respectively. The view is similar to that employed in Figure 2A.

The GHMP kinase family possesses three conserved motifs that create a network of interactions to stabilize the conformation of the catalytic center (Figures 4 and 5) [11,22]. In *Lm*MK, motif 1 is β1 through to a short α1. Residues on this motif create part of the substrate-binding site and side chains of Glu24 and His25 interact with the adjacent motif 3, which is in the C-terminal domain. Glu24 accepts hydrogen bonds donated from Lys279 NZ and the amide of Gly283, His25 donates a hydrogen bond to the carbonyl group of Thr283 (shown later). Functional groups associated with motif 3, such as the amide and hydroxyl groups of Thr283 could interact with the γ-phosphate of ATP. Motif 3 comprises residues on β8 and the hairpin bend to β9. Residues on one side of this motif interact with motif 1 as explained previously; on the other side there are interactions with components of motif 2. For example, Lys279 NZ and Ser281 OG participate in hydrogen bonds with the carbonyl group of Leu21 and amide of Ile20 respectively. Motif 2 comprises the N-terminal section of α3 and the preceding loop. Here the polypeptide conformation aligns several main chain amides, in conjunction with the α3 helix dipole, to bind the anionic tail of ATP in particular α- and β-phosphate groups. Some of the residues within these motifs are important for substrate binding and further details will follow.

### The ATP-binding site of *Lm*MK is distinct from related enzymes

**Figure 6:**
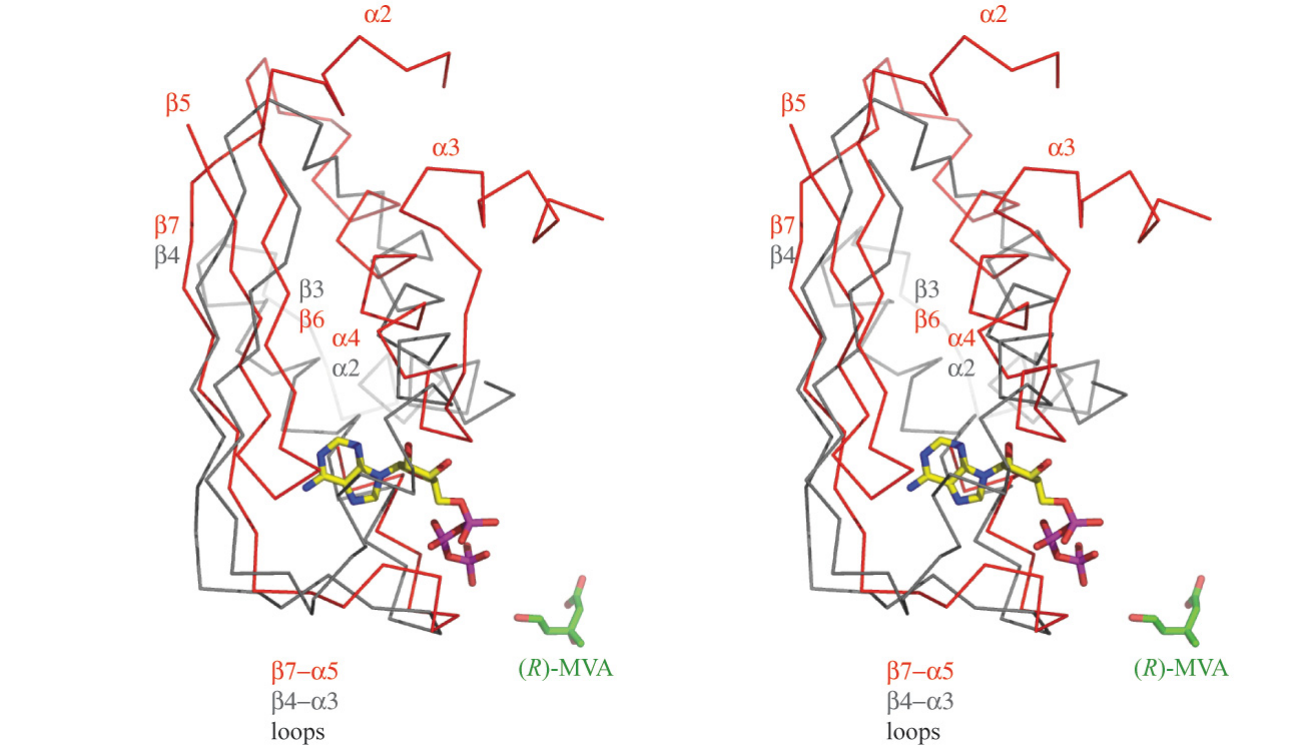
Cα-trace overlay for part of the N-terminal domains of the *Lm*MK and *Rn*MK structures. The Cα trace and labels for *Lm*MK are gray, and for *Rn*MK red. The substrate, (*R*)-MVA is from the *Lm*MK structure and shown as sticks colored green for C, red for O. The ATP (also in stick-mode, colored C yellow, N blue, O red, P purple) is from the *Rn*MK structure. Selected elements of secondary structure are labeled and colored according to the structure, grey *Lm*MK, red *Rn*MK.

A most striking difference in the arrangement of secondary structure elements in *Lm*MK compared to other GHMP kinases occurs in the N-terminal domain adjacent to the ATP binding site. This is best illustrated by the overlay of *Lm*MK and *Rn*MK, part of which is shown in Figure 6. The first four elements of *Lm*MK secondary structure (assigned as β1, α1, β2, β3) align well on the corresponding structural features of *Rn*MK (assigned by Fu *et al*., [9] as β1 and β2, α1, β3 and β4, β5). The structures then diverge as the mammalian MK sequence carries an insert forming a β-strand (β6) anti-parallel to β5, then an extended helix-loop-helix structure of α2, a disordered flexible segment and α3. A tight turn, in the vicinity of where adenine binds, then leads to α4. In *Lm*MK there is no strand equivalent to β6 (*Rn*MK). Strand β3, which is equivalent to β5 in *Rn*MK, is followed by a tight turn into α2. The helices α4 (*Rn*MK) and α2 (*Lm*MK) overlay well though in the parasite protein this helix is extended by two turns at the N-terminal end. The replacement of the insert and two helical segments in the rat enzyme with the short loop connecting β3-α2 in *Lm*MK results in a polypeptide conformation, not observed in structures of GHMP kinases, that lies across and restricts access to the ATP binding cavity (Figure 6). The detailed conformation of the β3-α2 loop in *Lm*MK may be influenced by contacts between symmetry related molecules. This loop is beside and forming hydrogen-bonding interactions with residues at the N-terminal end of β1, the C-terminal end of α3 and the α3–α4 loop of a symmetry-related molecule (not shown). Once into α2/α4 the *Lm*MK and *Rn*MK structures align well and then form a strand-loop-helix structure (motif 2, discussed earlier) that serves to create the base of the ATP-binding site (Figure 5). In *Lm*MK this is β4-loop-α3, in *Rn*MK β7-loop-α5.

In CDPME kinase [12] and HSK, [22,23] a helical insert also occurs before the α-helix equivalent to *Lm*MK α2, and similar to *Rn*MK, is placed to configure an open ATP binding site. Here, CDPME kinase and HSK carry residues that bind the ATP adenine in the less common *syn *conformation with respect to the ribose. In *Streptococcus pneumoniae *PMK [24] the polypeptide conformation also produces an open cavity into which ATP binds. In this case a segment of the polypeptide around the ATP-binding site is absent from the structural model due to disorder, and this implies a degree of conformational flexibility. Kinetic studies with *Enterococcus faecalis *MK indicate that GTP and CTP can also serve as substrates [25]. To investigate if such promiscuous substrate utilization also applied to *Lm*MK we tested the same compounds in the enzyme assay and also by co-crystallization in the absence and presence of (*R*)-MVA. In the latter case, to prevent possible turnover of the substrate we used the non-hydrolysable guanosine 5'-(β,γ-imido) triphosphate and CDP. Despite our efforts, extending to the complete structural analysis of several diffraction datasets (not shown), we were unable to obtain a binary structure of *Lm*MK in complex with ATP, any nucleotide, or a ternary dead-end complex.

To model ATP binding, the structures of *Lm*MK and the *Rn*MK:ATP binary complex were superimposed. In the *Rn*MK:ATP complex, the adenine is *anti *with respect to the ribose. The β and γ-phosphoryl groups together with Ser146 and Glu193 coordinate a Mg^2+^. In *Lm*MK the modeled adenine and ribose groups clash with the side chains of Tyr67, Lys71 and Glu74 at the N-terminal end of α2, Arg61 and Gln75 in the preceding loop region and Tyr147 in α4. These residues are positioned by a network of hydrogen bonding interactions, some mediated by water molecules. As discussed, the polypeptide conformation at the N-terminal segment of α2 and in the preceding loop is distinct from other MK structures and reduces access to the ATP binding site. The ATP-binding cavity in *Rn*MK, in contrast, is more open and the nucleotide is embedded into an environment with significant hydrophobic character. Conformational changes would have to occur, a number of hydrogen bonds would have to be disrupted and water molecules displaced to facilitate ATP binding by *Lm*MK as represented by the crystal structure.

The triphosphate moiety is placed to interact with the glycine-rich loop of the ATP-binding motif 2 in *Lm*MK. The β-phosphoryl group of ATP most likely interacts with Ser111 and Ser112, the latter a residue invariant in MK sequences, corresponding to Ser146 in *Rn*MK, the serine that participates in Mg^2+ ^coordination. The other residue involved in cation binding (Glu193 in *Rn*MK) is conserved (Glu144) in *Lm*MK and so conservation of the triphosphate positioning and interaction with MKs is likely. The model positions the γ-phosphoryl group in proximity to the strictly conserved catalytic residues Lys18 and Asp155 of *Lm*MK and the C5 hydroxyl group of substrate.

### Binding and recognition of mevalonate

**Figure 7:**
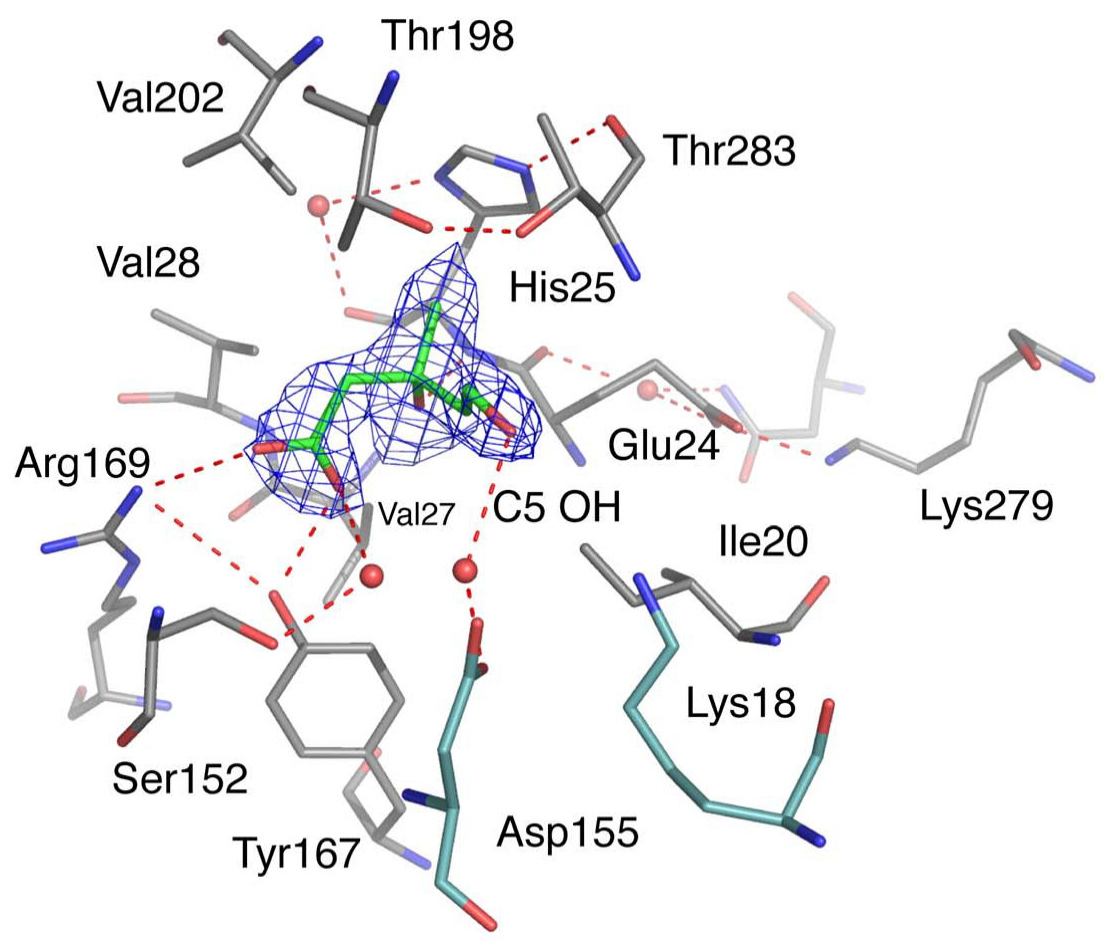
The substrate-binding site of *Lm*MK. Molecules are depicted in stick representation, all N positions are blue, O red. C atoms are grey except for (*R*)-MVA and the catalytic residues where they are green and cyan respectively. For the purpose of clarity only selected water molecules (red spheres) and hydrogen bonding interactions (red dashed lines) are shown. An omit difference density map (blue chicken wire) covering the substrate and the water molecule adjacent to the carboxylate group is shown. The map was calculated with coefficients |*F*o-*F*c|, α_calc _and contoured at 1.5 σ. *F*o and *F*c represent observed and calculated structure factor amplitudes respectively, α_calc _phases calculated on the basis of atomic coordinates of the model but not including the substrate or water oxygen.

(*R*)-MVA, binds in the deep cavity formed between the N-terminal and C-terminal domains, surrounded by residues on α1, the N-terminal end of α6, the C-terminal end of β1 and β5 and the α4–α5 loop (Figures 3, 4, 5, 6). The substrate carboxylate interacts with the guanidinium of Arg169, which is held in place by a hydrogen bond with the hydroxyl of Tyr167. This tyrosine also forms a hydrogen bond with the substrate carboxylate and is conserved both in terms of sequence and position in *Rn*MK (Tyr216). Sequence comparisons of MK from other species (not shown) reveal that Arg169, situated on a flexible loop between β5 and β6 is conserved in some species, e.g. in *Tb*MK and *Tc*MK. In other sequences this loop carries asparagine, glutamine, histidine and lysine residues, which would contribute a similar role in substrate binding. The carboxylate of (*R*)-MVA is also linked to the side chain of Ser152 via a water molecule. The side chains of Ile20, His25, Val27, Val28 and Val202 in *Lm*MK place (*R*)-MVA in the cavity by forming hydrophobic interactions with the substrate. The C3 hydroxyl group forms an intramolecular hydrogen bond to the carboxylate, which serves to stabilize the conformation of the substrate itself. This hydroxyl group accepts a hydrogen bond donated by the main chain amide of His25, a component of motif 1. The C3 methyl group is directed into a hydrophobic environment formed by the Cα of Thr283, the side chain of His25, Val28 and Val202. These interactions with the C3 substituents serve to discriminate for (*R*)-MVA over (*S*)-MVA. The C5 hydroxyl, the site of phosphorylation, is directed towards Lys18 NZ at a distance of approximately 4 Å, in addition a water molecule provides a bridge over to Asp155 and Thr198 (Figure 7).

Site-directed mutagenesis studies highlight the importance of the strictly conserved Glu24, His25 and Ser152 (*Lm*MK numbering) in MK. The replacement of the histidine by leucine or tyrosine significantly reduced activity of human MK whereas a lysine mutant did not fold correctly [17]. Houten *et al*. reported that a His25Pro mutant has no detectable MK activity [26]. In *Lm*MK, the side chain of His25 forms hydrogen-bonding interactions to its own main chain carbonyl, via a water molecule, and to the carbonyl group of Thr283, a residue in motif 3. In turn the side chain of Thr283 is held in place by a hydrogen bond with Thr198 (Figure 7). The preceding residue Glu24 participates in a salt bridge interaction with Lys279. Such interactions, help to force the main chain between Glu24 and His25 into a strained conformation, with a φ/ψ combination of 75/-45°, directing the amide group towards the substrate. The corresponding histidine and glutamate residues in *Mj*MK and *Rn*MK are located at nearly identical positions, but there is a peptide-flip compared to *Lm*MK so that the carbonyl not the main chain amide is directed towards the substrate-binding site. A peptide-flip does not accompany substrate binding since apo-*Lm*MK molecules retain the strained conformation. The conformation in other MK structures may be a consequence of analyses at medium resolution. The strictly conserved Glu24 is also important for stability. Mutations of the equivalent residue in human MK (Glu19Ala and Glu19Gln) destabilize the enzyme while a Glu19Asp change only slightly decreased activity [10]. The placement of an acidic side chain to interact with the nearby lysine (Lys330 in rat and human sequences) is therefore beneficial for function. Mutation of Ser201, equivalent to Ser152 in *Lm*MK, to alanine in human MK leads to a 100-fold decrease in binding affinity for substrate so proving an important role in substrate binding [16]. Ser152 OG also contributes to forming a hydrogen-bonding network, with the amide group of Ala154 and via water molecules, to stabilize the position of the catalytic Asp155 (not shown).

Fu *et al*. [9] and Yang *et al*. [8] modeled (*R*)-MVA into *Mj*MK and *Rn*MK respectively, and suggested a different binding mode compared to the experimentally determined complex reported here. They proposed, independently, that the substrate carboxylate interacts with an arginine (Arg241 or Arg201 in *Rn*MK and *Mj*MK respectively) and main chain amides contributed from motif 3. Although the guanidinium groups of Arg241 and Arg201 are similarly placed in the *Rn*MK and *Mj*MK structures, in *Lm*MK this is Ala196. The lack of conservation of an arginine at this position in MK sequences has already been discussed [18].

In this section, we have described the contributions and importance of 14 residues to the binding of substrate in the *Lm*MK:(*R*)-MVA complex either by direct or solvent mediated interactions, or by creating the binding site. These residues are depicted in Figure 7 together with selected hydrogen bonding interactions. Twelve of these residues are strictly conserved in mammalian MK sequences. The two exceptions are Arg169, which is equivalent to Gln218 and is a conservative change, and Thr283, equivalent to Ala334. This high degree of conservation in the substrate-binding site suggests that the substrate is bound to MKs in a similar conformation compared to (*R*)-MVA in *Lm*MK.

### The catalytic mechanism

**Figure 8:**
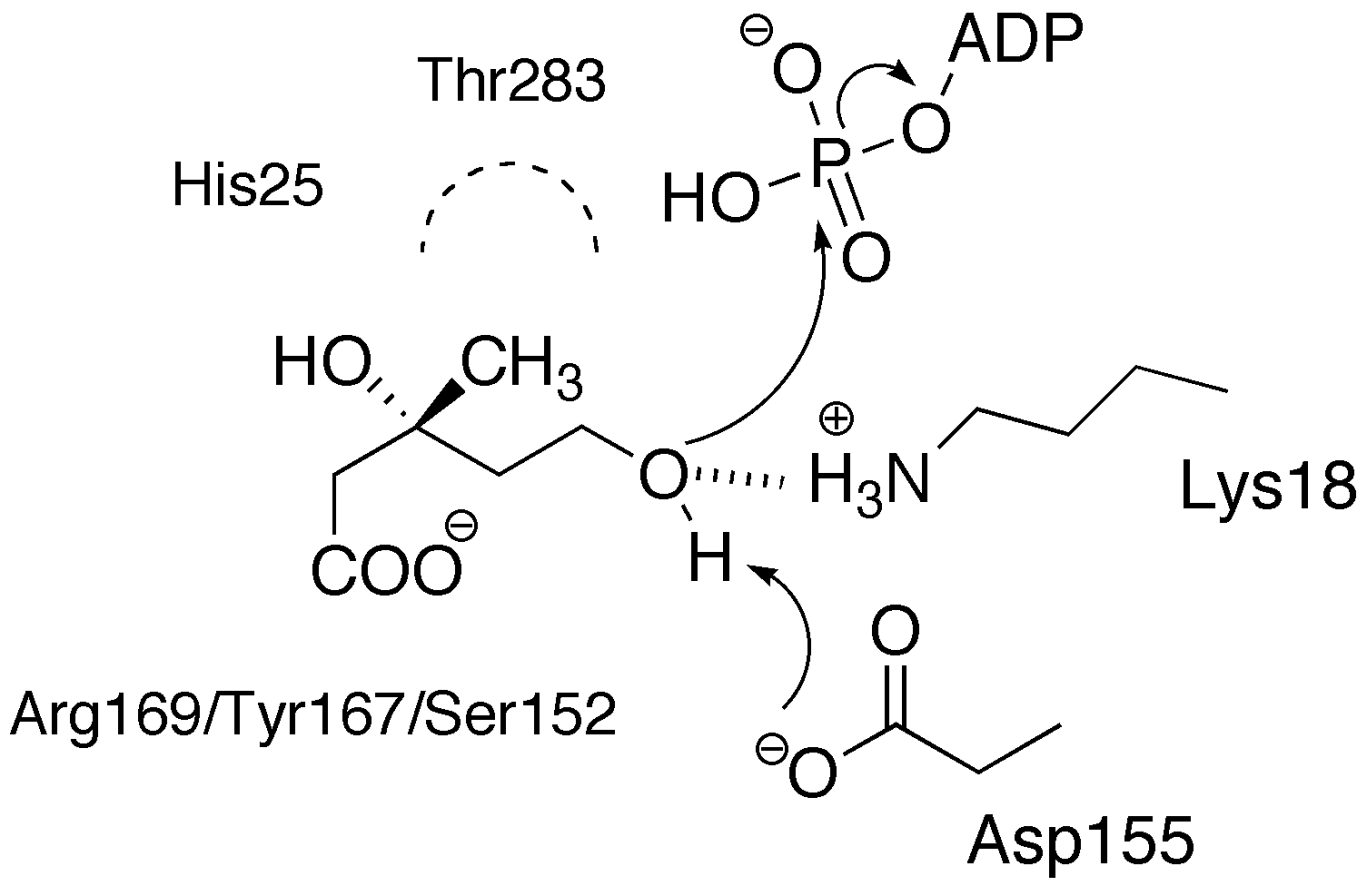
The proposed mechanism of catalysis and position of selected residues.

Knowledge of how (*R*)-MVA binds to *Lm*MK provides new information concerning the molecular recognition of substrate within the enzyme active site. In combination with biochemical and kinetic data, in particular that derived from human MK [10], this allows us to detail how the substrate is positioned and processed (Figures 7 and 8). MK carries out an ordered sequential reaction with mevalonate binding first, then ATP and, following catalysis the (*R*)-MVA 5-phosphate is released ahead of ADP Product inhibition by ADP is observed [27].

In the *Lm*MK:(*R*)-MVA complex, the substrate C5 hydroxyl group is directed towards the side chains of Lys18 (~4 Å) and Asp155 (~5 Å) as well as the γ-phosphoryl of the modeled ATP (~6 Å). The positions of the strictly conserved lysine/aspartate pair are nearly identical in *Lm*MK, *Mj*MK and *Rn*MK. In kinetic studies of human MK, no significant activity of the Asp155Ala and Asp155Asn mutants was observed and a replacement of the basic Lys18 by methionine resulted in a 56-fold decrease of V_max _in *Rn*MK [15]. Asp155 acts as a catalytic base and abstracts the proton from the C5 hydroxyl group of (*R*)-MVA to generate a potent nucleophilic alkoxide. The *p*Ka of the C5 hydroxyl group must be lowered in order for proton abstraction to occur, a process required to generate a nucleophilic alkoxide. Presumably the basic Lys18 is primarily responsible for lowering the *p*Ka and perhaps also stabilizing the alkoxide. Once the ATP is in place then the C5 alkoxide will attack and acquire the γ-phosphoryl group. A pentacoordinate transition state is likely and may be stabilized by Lys18 and the presence of a divalent cation, as commonly observed in many kinases.

## Conclusion

Our study reveals, for the first time, MK activity in trypanosomatids. High-resolution crystal structures of *Lm*MK have been determined and provide the first experimentally derived model for any MK in complex with mevalonate. The structure reveals how this substrate binds, the structural basis for chiral discrimination of (*R*)- against (*S*)-form MVA and is consistent with previously published biochemical data regarding mechanism. The enzyme, not surprisingly displays the GHMP kinase fold and structural overlay indicates close similarities to *Rn*MK for most of the structure. However, comparisons reveal a significant difference at the ATP-binding site that may help explain the low activity of recombinant *Lm*MK and our inability to obtain structural information on an ATP complex. Sequence comparisons suggest that the β3-α2 loop and α2 segments, including many of the hydrogen bonding interactions in *Lm*MK are conserved in other trypanosomatid enzymes, *Tb*MK and *Tc*MK. As *Lm*MK and *Tb*MK expressed in procyclic-form *T. brucei *exhibit a greatly enhanced enzyme activity compared to the recombinant *Lm*MK, it is possible that some parasite specific factor may influence the catalytic reaction and further studies are required to investigate such a hypothesis.

The high degree of conservation between trypanosomatid and mammalian MKs suggests that it would be difficult to develop a small molecule that would selectively inhibit the parasite enzyme by interacting within the mevalonate-binding site. The differences observed in the ATP-binding sites might be exploitable for structure-based inhibitor design but a decision would best be taken when a structure of a trypanosomatid MK in complex with ATP has been determined.

## Methods

### Cloning, expression, purification of *Lm*MK for biophysical characterization

The putative gene encoding *Lm*MK (UniProt entry Q4Q6K7) was isolated from genomic DNA by PCR with forward 5'-CATATGTCTAAGCCCGTCAAGAGC-3' and reverse 5'-CTCGAGTTAT AGGTTCGACGCGGCG-3' primers containing *Nde*I and *Xho*I restriction sites respectively (underlined). The gene was cloned into the pCR blunt II TOPO vector (Invitrogen) then subcloned into the pET15b vector (Novagen). The resulting plasmid was transformed into *E. coli *BL21 (DE3) (Stratagene). The His-tagged protein was produced overnight at 30°C in LB medium containing ampicillin (100 mg/L) after induction with 1 mM isopropyl-β-D-thiogalactopyranoside. Cells, suspended in 50 mM Tris·HCl pH 8.5, 250 mM NaCl and 3 mM β-mercaptoethanol, were lysed at a pressure of 25 Kpsi (One Shot, Constant Cell Disruptions Systems) and the extract clarified by centrifugation (20,000 g, 30 min, 4°C). The supernatant was loaded onto a nickel chelating chromatography column (GE Healthcare) and eluted with a linear gradient from 70 to 800 mM imidazole. The His-tag was removed from *Lm*MK by digestion with thrombin for 6 h at 4°C. After dialysis against 50 mM Tris·HCl pH 7.7, 50 mM NaCl and 1 mM DTT, the enzyme was purified further by anion exchange chromatography and pooled fractions dialyzed against 10 mM Tris·HCl pH 8.5, 20 mM NaCl and 1 mM DTT. The protein was concentrated by centrifugation with a VivaSpin 20 MW cutoff 10,000 Da (Vivascience) and the protein concentration determined spectrophotometrically using an extinction coefficient of 28,000 M^-1 ^cm^-1 ^(280 nm).

For the production of SeMet *Lm*MK, the plasmid was transformed into *E. coli *strain B834 (Stratagene). Bacteria were grown in M9 medium, which was supplemented with 4 mg/L FeSO_4_·7H_2_O and 0.5 g/L each of adenine, guanosine, thymine and uracil. In addition, 40 mg/L of the usual amino acids except methionine, which was replaced by 100 mg/L L-SeMet (Sigma-Aldrich), were added. Protein production and purification was performed according to the protocol described for the native enzyme. Full incorporation of SeMet was confirmed by matrix-assisted laser desorption/ionisation time-of-flight mass spectrometry (data not shown).

### Cloning of *T. brucei *and *L. major *MK genes for ligation into the pLew 82 vector

Putative MK genes were identified in *T. brucei *(entry Q4Q6K7 in the UniProt database) and *L. major *genome databases (see above) in advance of annotation (Sanger Centre) using the *S. cerevisiae *gene sequence as the query. The open reading frame encoding *Tb*MK was amplified from *T. brucei *genomic DNA using forward and reverse primers 5'-GAGGAGAAGCTTATGCACGTGGCTGTTAAGGAC-3' and 5'-TGCTTAATTAATAGCTTACTTCCGCCGGGCTG-3' containing *Hin*dIII and *Pac*I restriction sites respectively (underlined). The open reading frame encoding *Lm*MK was amplified from *L. major *genomic DNA using the forward and reverse primers 5'- GAGGAGAAGCTTATGTCCGTTTTTTTCGCTGTGACT-3' and 5'- TGCTTAATTAATAGGTTCGACGCGGCGGACGGCTG-3' containing *Hin*dIII and *Pac*I restriction sites respectively (underlined). Bands of the expected size of ~ 1 Kb for both genes were amplified using *Pfu *polymerase, purified (QIAquick PCR purification kit, Qiagen) and cloned into pC*R*-Blunt II TOPO (Invitrogen). The ORFs were excised then ligated into the tetracycline inducible expression vector pLew82 [28], which integrates a C-terminal HA-epitope tag.

### Cultivation and genetic modification of *T. brucei*

Procyclic form *T. brucei *strain 427, previously modified to express T7 polymerase and the tetracycline repressor protein, were grown in SDM-79 media supplemented with 5% sodium bicarbonate and the appropriate drug selection, at 28°C with 5 % CO_2 _as described elsewhere [29,30]. Mid-log cells were electroporated with 50 μg of *Not*1-linearized pLew82 plasmids in a total volume of 400 μL of cytomix buffer. Transfected parasites were selected in medium containing phleomycin (2.5 μg/mL) to obtain the cell lines *TbMK-HA*^Ti^, and *LmMK-HA*^Ti^. When tetracycline was added to the media to induce over-expression, a final concentration of 1 μg/mL was used. Cells were counted each day and passaged when the density was between 2 and 3 × 10^6 ^(normally every second day).

### Over expressing *T. brucei *and *L. major *MK genes in procyclic form *T. brucei*

Mid-log *T. brucei TbMK-HA*^Ti^, *LmMK-HA*^Ti ^and wild type procyclic form cells which had been grown in the presence of tetracycline for one day, were harvested by centrifugation (800 g, 10 min), the cell pellets were washed in TDB buffer (25 mM KCl, 400 mM NaCl, 5 mM MgSO_4_, 100 mM Na_2_HPO_4_, NaH_2_PO_4_, pH 7.4, 100 mM glucose) and used either for western blotting or immunoprecipitation (Figure 2). For western blotting the cells (2 × 10^6^) were lysed and denatured directly in hot sample buffer and run on an SDS/10 % polyacrylamide gel and transferred to an ECL-Nylon membrane (Amersham). After blocking overnight in PBS-5 % skim milk powder, protein was detected using the primary monoclonal antibody; rat anti-HA (Roche), followed by a secondary horseradish peroxidase conjugated rabbit anti-rat immunoglobulin (Jackson) and ECL western detection reagents (Amersham). For immunoprecipitation, cells (1 × 10^8^) were lysed in 1 mL of Tris-HCl (20 mM pH 7.0), NaCl (0.15 M) and NP-40 (1%). After agitating for 30 min the cell ghosts were spun down and the supernatant added to 100 μL of equilibrated anti-HA affinity matrix (Roche) and agitated at 4°C overnight. The beads were spun down and washed twice with 500 μL of lysis buffer and resuspended in 100 μL of potassium phosphate buffer (0.1 M) and stored at -20°C until required.

### MK assays and binding studies

A coupled spectroscopic assay was performed according to a published protocol with varying concentrations of recombinant *Lm*MK; *Mj*MK provided the positive control [18]. Briefly, in a 1 mL cuvette, 0.1 M Tris·HCl pH 7.5, 5 mM MgCl_2_, 0.5 M phosphoenolpyruvate, 0.1 M NADH, 0.1 M ATP, 0.1 M (*R/S*)-mevalonic acid, 40 U pyruvate kinase and 27.5 U lactate dehydrogenase were incubated at 25°C. The enzyme activity was monitored at 340 nm for 300 seconds (Shimadzu UV-1601).

In a radioactive assay, 0.1 M KH_2_PO_4_/K_2_HPO_4 _pH 7.0, 0.1 M ATP, 10 μM reduced glutathione, 5 μM MgCl_2_, 833 μM [^3^H] (*R*)-MVA (60 Ci/μmol) (ARC) with either 31.0 μM *Lm*MK (approximately 115 μg) or with 20 μM of immunoprecipitate α-HA *Lm*MK or *Tb*MK (equivalent to 2 × 10^7 ^procyclic cells) in a volume of 100 μL were incubated for 2 h at 30°C. The reaction was quenched by heat inactivation at 100°C for 2 min, the protein was spun down and the supernatant applied to a 30 cm strip of Whatman paper. The pellet was mixed with 20 μL water, centrifuged and the washing added to the Whatman paper. Unreacted [^3^H] (*R*)-MVA was separated from the [^3^H] (*R*)-MVA 5-phosphate product by descending thin-layer chromatography using propan-1-ol:NH_3_:H_2_O (6:2:1). The same amount of [^3^H] (*R*)-MVA provided a negative control. [^3^H] (*R*)-MVA and [^3^H] (*R*)-MVA 5-phosphate were localized using a Bioscan A*R*-200 linear analyzer at a distance of 4 cm or 23.5 cm from the origin, respectively. The Whatman paper was cut into 1 cm strips and the radioactivity per strip quantified by liquid scintillation counting. The combined counts corresponding to [^3^H] (*R*)-MVA 5-phosphate were then compared with the initial amount of [^3^H] (*R*)-MVA utilized in the enzymatic reaction.

The potential association of TNP-ATP (Molecular Probes) with *Lm*MK was investigated following a published protocol [19]. Measurements were carried out in 100 mM Tris-HCl pH 7.0, 100 mM NaCl and 10 mM MgCl_2 _at enzyme concentrations of 3.2 μM or 12.8 μM, respectively. TNP-ATP concentration was varied between 15 μM and 152 μM. For measurements, (Varian Cary Eclipse Fluorescence Spectrometer) the excitation wavelength was 408 nm and the emission spectra obtained by scanning 500 to 600 nm. The positive control was binding of TNP-ATP to *T. brucei *MDD (data not shown).

### Quaternary structure investigation

The possibility of an oligomeric assembly was investigated by gel filtration and analytical ultracentrifugation. The gel filtration column HiLoad 16/60 Superdex™ 200 prep grade (GE Healthcare) was calibrated with the Gel Filtration LMW Calibration Kit (GE Healthcare). The *Lm*MK eluted from the column at a volume of 92 ml, which corresponds to a molecular mass of approximately 35 kDa (data not shown).

A sample of *Lm*MK, (0.5 mg/mL in 10 mM Tris·HCl pH, 20 mM NaCl and 1 mM *tris*(2-carboxyethyl)phosphine hydrochloride) was used in sedimentation velocity experiments performed at a wavelength of 280 nm, at 45,000 rpm and 20°C, using a Beckman Coulter XL-i analytical ultracentrifuge. The sample was centrifuged with A_280 _measured every 5 min over a period of 15 hours. The resultant data were analysed using the program *SEDFIT *[31]. The sedimentation co-efficient that was obtained, 2.97 s, corresponds to a mass of approximately 33.6 kDa.

### Crystallographic methods

#### Crystallization

*Lm*MK crystallized using hanging drop vapor diffusion at 18°C. Drops were assembled from 2 μL reservoir (1.15 M sodium citrate pH 6.2) and 2 μL protein solution (7.5 mg/mL protein, 3 mM AMPPNP and 6 mM (*R/S*)-MVA). The enantiomeric mixture of the substrate was prepared from *RS*-mevalonolactone (Sigma-Aldrich) according to literature methods [32]. Thin monoclinic plates (0.4 mm × 0.2 mm × < 0.1 mm) grew after approximately one week. Crystals of the native protein in complex with (*R*)-MVA formed under similar conditions with 25 mM AMPPNP, 50 mM (*R/S*)-MVA and 10 mM MgCl_2 _in the drop.

#### Data collection

A crystal of SeMet *Lm*MK was flash cooled directly in a stream of gaseous nitrogen at 100 K and diffraction data were measured on beam-line ID14-4 at the European Synchrotron Radiation Facility (Grenoble, France). A fluorescence scan was used to determine the Se K absorption edge wavelength for data collection, λ = 0.97945 Å, and data recorded using a Q315r ADSC CCD detector. Data for the binary substrate complex were collected using a Rigaku 007 Micromax rotating-anode generator (Cu K_α_, λ = 1.5418 Å) operating at 30 mA and 40 kV, coupled to a R-AXIS IV^++ ^dual image plate system. All data were processed and scaled with *Denzo/Scalepack *[33].

#### Structure determination

We were unable to solve the *Lm*MK structure by molecular replacement and therefore adopted a single wavelength anomalous dispersion approach. Data to 2.0 Å identified six Se positions out of eight (*SOLVE*[34]) and the correct enantiomorph gave a figure-of-merit of 0.38 and a Z-Score of 40. After density modification (RESOLVE [35]) the figure-of-merit increased to 0.72 with a correlation coefficient of 0.75. Automated model building (ARP/wARP [36]) constructed an initial model of 531 (out of 658) residues and the structure was refined (*REFMAC5 *[37]) to an *R-*factor/*R*-work of 26.2 % and an *R-*free of 32.4 % of 1.75 Å employing strict NCS restraints. 5% of the data were set aside for the calculation of *R-*free. Additional residues and water molecules were placed manually into the electron density with *COOT *[38] interspersed by refinement with *REFMAC5*. Towards the end of the refinement, the NCS restraints were released. The quality of the structure was assessed with *PROCHECK *[39]. All residues are situated in most favored or additionally allowed regions of the Ramachandran plot apart from His25 in both molecules. Three residues at the C-terminus are disordered and not included in the structure.

The crystal structure of SeMet *Lm*MK was used in molecular replacement calculations (*MOLREP *[40]) to provide the initial model for the substrate complex. The correlation coefficient of the first model was 0.67 and *R-*work 33.1 %. The electron density clearly indicated the presence of (*R*)-MVA in molecule A of the asymmetric unit. The refinement process was completed in a similar fashion to SeMet *Lm*MK. Statistics are presented in Table 1. Figures were prepared with *Chemdraw *(Adept Scientific), *PyMOL*^§ ^and *ALINE *(C. S. Bond, personal communication). Coordinates and structure factors have been deposited in the Protein Data Bank [PDB:2HFS and PDB:2HFU].

### Footnotes

^# ^Z-score measures the statistical significance of the best alignment and typically, dissimilar structures present a Z-score less than 2.0.

^§ ^DeLano, W. L. (2002) The PyMOL Molecular Graphics System, DeLano Scientific, San Carlos, CA, USA.

## Abbreviations

AMPPNP, adenosine 5'-(β,γ-imido) triphosphate; CDPME, 4-diphosphocytidyl-2*C*-methyl-D-erythritol; DMAPP, dimethylallyl pyrophosphate; DOXP, deoxyxyulose phosphate; GHMP, galacto/homoserine/mevalonate/phosphomevalonate kinase; GK, galactokinase; HMG-CoA, 3-hydroxyl-3-methylglutaryl-coenzyme A; HSK, homoserine kinase; IPP, isopentenyl pyrophosphate; MDD, mevalonate 5-diphosphate decarboxylase; MK, mevalonate kinase; *Lm*MK, *Leishmania major *mevalonate kinase; *Mj*MK, *Methanococcus jannaschii *mevalonate kinase; MVA, mevalonate; NCS, non-crystallographic symmetry; PDB, Protein Data Bank; PMK, phosphomevalonate kinase; r.m.s.d., root mean square deviation; *Rn*MK, *Rattus norvegicus *mevalonate kinase; SAD, single-wavelength anomalous dispersion; SeMet, selenomethionine; *Tb*MK, *Trypanosoma brucei *mevalonate kinase; *Tc*MK, *Trypanosoma cruzi *mevalonate kinase; TNP-ATP, 2'(3')-O-(2,4,6-trinitrophenyl) adenosine 5'-triphosphate.

## Authors' contributions

TS carried out biochemical and crystallographic experiments, TKS carried out all work with parasites, and together TS and TKS carried out enzyme assays. WNH conceived of the study, and participated in its design and coordination. All authors were involved in data interpretation and in writing the manuscript.

## Figures and Tables

**Table 1:** Crystallographic statistics

	SeMet *Lm*MK	*Lm*MK:*R*-MVA complex
Unit-cell parameters		
*a*, *b*, *c *(Å),	41.3, 88.5, 88.2	41.2, 88.4, 87.7
β (°)	103.6	103.5
Space group	*P*2_1_	*P*2_1_
Resolution range (Å)	40.0-1.75	40.0-1.9
Observations/redundancy	120,161/3.4 (3.2)^a^	43,849/4.0 (3.2)
Completeness (%)	97.2 (92.6)	91.2 (65.4)
Mosaicity (°)	0.5	1.0
<I/σ(I)>	20.1 (2.9)	15.3 (2.8)
*R-*merge (%)^b^	5.6 (41.1)	7.3 (44.4)
*R*-work/*R*-free(%)^c^	21.7/27.7	17.6/23.0
r.m.s.d. bond lengths (Å)	0.011	0.006
r.m.s.d. bond angles (°)	1.227	0.952
Wilson *B *value (Å^2^)	16.3	22.1
Number of atoms		
Protein	5026	5157
*R*-MVA		10
Solvent	590	695
Average *B *values (Å^2^)		
Protein	16.5	21.1
*R*-MVA		26.0
Solvent	24	31.9
Ramachandran plot		
Most favored (%)	94.6	94.6
Additional allowed (%)	5.0	5.0
Disallowed region (%)^d^	0.4	0.4

^a ^Numbers in parenthesis refer to an outer resolution bin of width approximately 0.1 Å.

^b ^*R*-merge = Σ_*h*_Σ_*i*_|I(*h,i*) -<I(*h*)>|/Σ_*h*_Σ_*i *_I(*h*,*i*), where I(*h,i*) is the intensity of the *i*th measurement of reflection *h *and <I(*h*)> is the mean value of I(*h,i*) for all *i *measurements.

^c ^*R*-work = Σ_*hkl*_||*Fo*|-|*Fc*||Σ|*Fo*|, where *Fo *is the observed structure-factor amplitude and *Fc *the structure-factor amplitude calculated from the model. *R*_free _is the same as *R*_work _except only calculated using a subset, 5%, of the data that are not included in any refinement calculations.

^d ^His25 in all molecules.
